# Ultrasound Normalized Weighted Cumulative Residual Entropy Imaging: A New Quantitative Ultrasound Method for Characterizing Hepatic Steatosis

**DOI:** 10.3390/diagnostics16193253

**Published:** 2026-10-08

**Authors:** Huilin Si, Ruiyang Gao, Dar-In Tai, Po-Hsiang Tsui, Zhuhuang Zhou

**Affiliations:** 1 School of Computer and Artificial Intelligence, Beijing Technology and Business University, Beijing 100048, China; sihuil@th.btbu.edu.cn; 2 Department of Biomedical Engineering, College of Chemistry and Life Science, Beijing University of Technology, Beijing 100124, China; gaoruiyang@emails.bjut.edu.cn; 3 Department of Gastroenterology and Hepatology, Chang Gung Memorial Hospital at Linkou, Chang Gung University, Taoyuan 333423, Taiwan; tai48978@cgmh.org.tw; 4 Department of Biomedical Engineering and Environmental Sciences, National Tsing Hua University, Hsinchu 300044, Taiwan

**Keywords:** quantitative ultrasound, ultrasound backscatter, envelope statistics, cumulative residual entropy, weighted cumulative residual entropy, cumulative distribution function, ultrasound tissue characterization, ultrasound radiofrequency signal, hepatic steatosis

## Abstract

**Background/Objectives:** In this paper, we propose ultrasound normalized weighted cumulative residual entropy (NWCRE) imaging, a novel quantitative ultrasound (QUS) technique derived from the information-theoretic weighted cumulative residual entropy (WCRE) for hepatic steatosis characterization. **Methods:** The proposed ultrasound NWCRE imaging method adopted cumulative distribution function (CDF)-driven non-parametric estimation, and parallel computing was implemented to improve computational efficiency. For a comparison study, the previously reported ultrasound normalized cumulative residual entropy (NCRE) imaging was also implemented. We further mathematically derived the finite upper bound of integrals for both NWCRE and NCRE to convert improper integrals into proper integrals for numerical calculation. Phantom simulations with varied scatterer number densities and two groups of clinical datasets for hepatic steatosis assessment were investigated for validation. **Results:** Phantom results demonstrated that both NWCRE and NCRE values decreased with increasing scatterer concentrations. For clinical group A (72 liver donors; magnetic resonance spectroscopy-measured hepatic fat fraction (HFF) served as the reference), NWCRE imaging exhibited a slightly stronger correlation with log_10_(HFF) (Spearman *r* = −0.6749, *p* < 0.0001) than NCRE (Spearman *r* = −0.6695, *p* < 0.0001). For clinical group B (204 chronic hepatitis B patients; liver biopsy served as the histopathological ground-truth reference), NWCRE imaging yielded comparable diagnostic performance to NCRE for discriminating hepatic steatosis grades (all *p* > 0.05), with area under the receiver operating characteristic curve (AUC) values of 0.78, 0.85 and 0.86 for ≥G1, ≥G2 and ≥G3 steatosis, respectively. Across phantom simulations and both clinical groups, the dynamic ranges of NWCRE imaging were statistically significantly higher than those of NCRE imaging (all *p* < 0.0001). **Conclusions:** The proposed parallelized ultrasound NWCRE imaging delivers diagnostic performance comparable to NCRE imaging while improving the dynamic range over NCRE imaging, providing a potential CDF-based QUS tool for quantitative evaluation of hepatic steatosis.

## 1. Introduction

Hepatic steatosis is a key manifestation of metabolic dysfunction-associated fatty liver disease (MAFLD), which affects approximately 30% of the global population and constitutes a major contributor to the global burden of chronic liver disease [1]. Liver biopsy remains the gold standard modality for the diagnosis of hepatic steatosis. Nevertheless, it is an invasive procedure associated with risks of sampling bias and procedural complications.

Ultrasonography serves as a first-line modality for evaluating and monitoring hepatic steatosis. However, conventional B-mode ultrasound only enables qualitative assessment, relying exclusively on the envelope amplitude of backscattered ultrasound signals. Quantitative ultrasound (QUS), by contrast, extracts quantitative frequency, phase, and statistical features from ultrasound backscatter radiofrequency (RF) signals [2,3,4,5], acting as a complementary technique to standard B-mode ultrasonography.

Ultrasound backscatter envelope statistics parametric imaging constitutes a key category of QUS [2,3,4,5]. It can be broadly classified into model-based and model-free methods. Model-based approaches, exemplified by ultrasound Nakagami imaging [6,7,8,9] and homodyned-K imaging [10,11,12,13], are built upon generalized statistical models of backscatter envelopes. In contrast, ultrasound entropy imaging quantifies the information-theoretic entropy of backscatter envelopes without assuming any specific statistical distribution [14,15,16,17,18], representing a rapidly evolving direction within QUS research.

The existing literature indicates that ultrasound Shannon entropy imaging represents the predominant entropic framework for biological tissue characterization, with histogram-based probability distribution function (PDF) estimators serving as the primary tool for ultrasound Shannon entropy estimation [14,15,16,17,18]. Nevertheless, the performance of conventional histogram-based entropy imaging is susceptible to histogram configuration parameters, particularly the bin count. To overcome these limitations of PDF-based Shannon entropy imaging, our previous work has introduced the information-theoretic cumulative residual entropy (CRE) into the QUS field and developed cumulative distribution function (CDF)-based ultrasound normalized CRE (NCRE) imaging [19]. However, this ultrasound NCRE imaging suffers from limited dynamic range.

Weighted CRE (WCRE) is known to be a variant of CRE in information theory [20,21], yet it has not been incorporated into QUS. In this study, we aimed to introduce WCRE into QUS and proposed ultrasound-normalized WCRE (NWCRE) imaging. We hypothesized that ultrasound NWCRE imaging is capable of hepatic steatosis characterization and might possess a higher dynamic range than NCRE imaging. To validate these hypotheses, we performed phantom simulations and clinical hepatic steatosis experiments to collect ultrasound RF signals of various scatterer concentrations (number densities) and hepatic steatosis grades, which were used to construct NWCRE images, with a comparison to NCRE images. In addition, we incorporated the parallelized computation technique [19] into the proposed ultrasound NWCRE imaging. The feasibility of NWCRE imaging in characterizing various scatterer number densities and steatosis grades was demonstrated, with a statistically significantly higher dynamic range than NCRE imaging. The main contributions of this study are as follows:

(1) To the best of our knowledge, this study is the first to integrate WCRE into QUS and to propose ultrasound NWCRE imaging, which mitigates the drawback of NCRE imaging as a limited dynamic range.
(2) The parallelized computation technique is incorporated to improve the efficiency of the proposed algorithm.
(3) Phantom simulation experiments and clinical experiments on hepatic steatosis assessment are performed.

## 2. Materials and Methods

**Figure 1:**
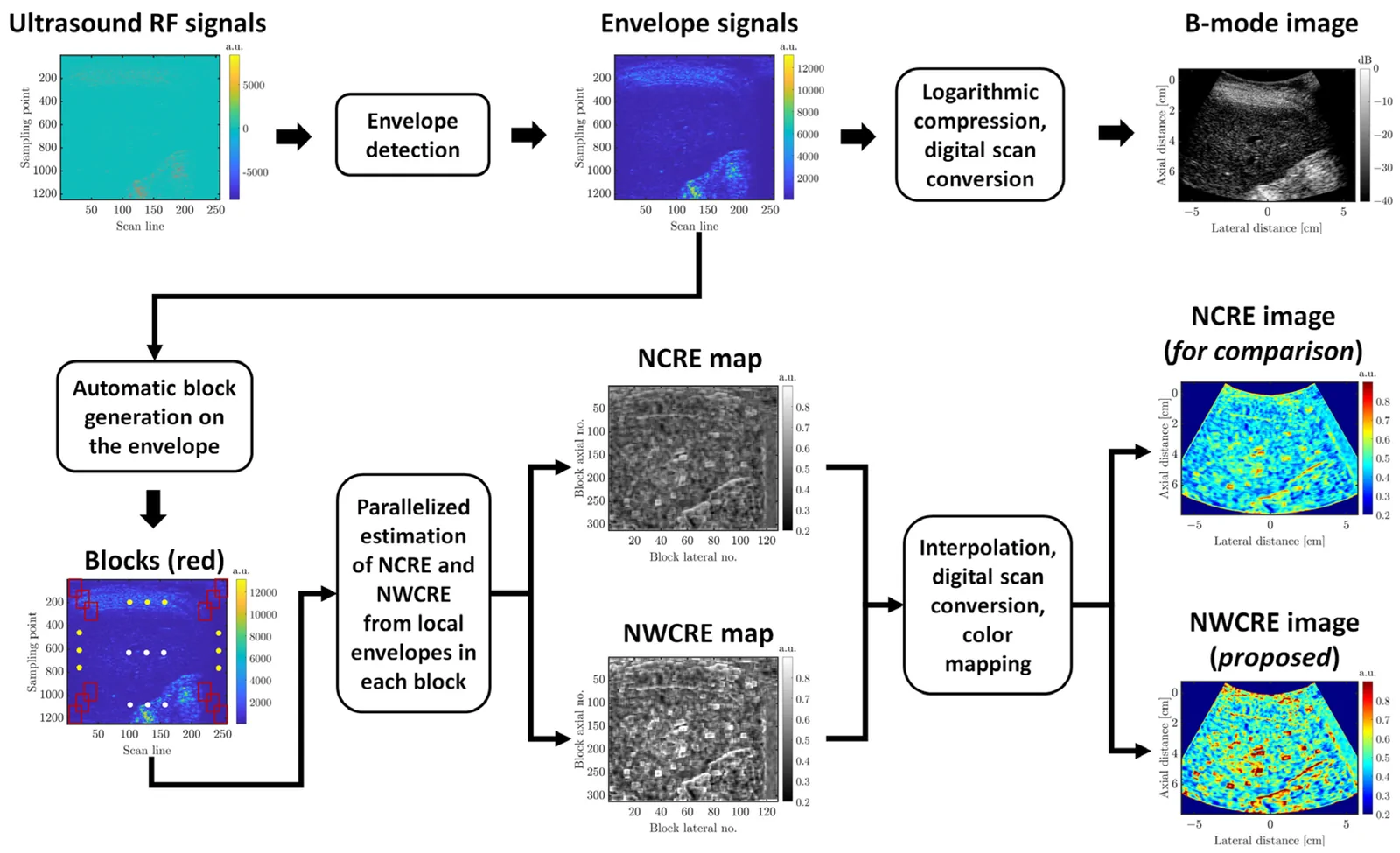
Algorithmic flow chart of the proposed ultrasound normalized weighted cumulative residual entropy (NWCRE) imaging method for characterizing hepatic steatosis. RF: radiofrequency; NCRE: normalized cumulative residual entropy.

Figure 1 shows the algorithmic flow chart of the proposed ultrasound NWCRE imaging method for characterizing hepatic steatosis, with NCRE imaging included for comparison purposes. In the subsequent subsections, the theoretical background of ultrasound NCRE is presented in Section 2.1. The theory and methodology of the proposed ultrasound NWCRE and its parametric imaging are described in Section 2.2 and Section 2.3. Collections of ultrasound RF signals in phantom simulations and clinical experiments are detailed in Section 2.4 and Section 2.5. Statistical analysis procedures are provided in Section 2.6.

### 2.1. Ultrasound Normalized Cumulative Residual Entropy

Let **X** denote an *N*-dimensional random vector, **X**∈ℝ*^N^* and X≠0, and let *F*(.) denote the CDF of **X**. In information theory, unlike the classical Shannon entropy, which is based on the PDF of **X**, the cumulative residual entropy (CRE) is a CDF-based metric for quantifying the uncertainty of **X** [22]:

(1) $$\mathrm{CRE}=-\int_{0}^{\infty }S(u)\ln[S(u)]du$$

where *u* is an integration variable and *S*(*u*) is the survival function (SF), *S*(*u*) = 1 − *F*(*u*).

Our recent work [19] addressed the research question of how to introduce the information-theoretic CRE into the QUS field to measure the backscatter envelope signal uncertainty or complexity. Let **A** denote an *N*-dimensional ultrasound backscatter envelope vector, $A\in {\mathbb{R}}_{+}^{N}$ and A≠0. First, **A** was normalized by its root mean square (RMS) value to eliminate the influence of mean echo intensity:

(2) $$A^{\prime }=\frac{A}{\sqrt{E[A^{2}]}}$$

where $A^{\prime }$ is the normalized ultrasound envelope vector, and *E*[.] denotes the expectation operator. Let $F^{\prime }(.)$ and $S^{\prime }(.)$ denote the CDF and SF of $A^{\prime }$, respectively, $S^{\prime }(.)=1-F^{\prime }(.)$. Then, ultrasound NCRE of **A** was obtained by

(3) $$\mathrm{NCRE}=-\int_{0}^{\infty }S^{\prime }(u)\ln[S^{\prime }(u)]du$$

Our previous work demonstrated that ultrasound NCRE decreases as the local tissue scatterer concentrations (number densities) increase and that it is feasible for ultrasound tissue characterization [19].

### 2.2. Ultrasound Normalized Weighted Cumulative Residual Entropy

In information theory, weighted cumulative residual entropy (WCRE) is a variant of CRE [20,21]:

(4) $$\mathrm{WCRE}=-\int_{0}^{\infty }uS(u)\ln[S(u)]du$$

However, WCRE has not been introduced into QUS. In this study, we proposed the ultrasound NWCRE of the backscatter envelope vector **A**:

(5) $$\mathrm{NWCRE}=-\int_{0}^{\infty }uS^{\prime }(u)\ln[S^{\prime }(u)]du$$

Equation (5) shows that NWCRE is an improper integral from zero to infinity. As such, the following question can be raised: Is it possible to narrow the upper limit of integration rather than infinity? Since the SF $S^{\prime }(.)$ is a function of the normalized envelope vector $A^{\prime }$, we sought to determine the upper limit of $A^{\prime }$, i.e., the maximum of $A^{\prime }$, ${A^{\prime }}_{\max}$. From Equation (2), it is derived that

(6) $$A^{\prime }=\frac{A}{\sqrt{E[A^{2}]}}=\frac{A}{\sqrt{\frac{1}{N}\sum_{i=1}^{N}A_{i}^{2}}}=\frac{\sqrt{N}A}{\sqrt{\sum_{i=1}^{N}A_{i}^{2}}}$$

where the subscript *i* denotes the *i*th element of **A**. Because

(7) $$\sum_{i=1}^{N}A_{i}^{2}=A_{k}^{2}+\sum_{i=1,i\neq k}^{N}A_{i}^{2}$$

where the subscript *k* denotes the *k*th element of **A**, and because

(8) $$\sum_{i=1,i\neq k}^{N}A_{i}^{2}\geq 0$$

we obtained

(9) $$A_{k}\leq \sqrt{\sum_{i=1}^{N}A_{i}^{2}}$$

Substituting Equation (9) into Equation (6) yields

(10) $${A^{\prime }}_{k}=\frac{\sqrt{N}A_{k}}{\sqrt{\sum_{i=1}^{N}A_{i}^{2}}}\leq \sqrt{N}$$

which gives the upper limit of $A^{\prime }$ as $\sqrt{N}$, i.e., ${A^{\prime }}_{\max}$ = $\sqrt{N}$. This shows another benefit of the normalization, making the normalized envelope vector $A^{\prime }$ have a maximum only related to the number of samples *N*. With Equation (10), the improper integral in Equation (5) can be turned into a proper integral:

(11) $$\mathrm{NWCRE}=-\int_{0}^{\sqrt{N}}uS^{\prime }(u)\ln[S^{\prime }(u)]du$$

The same findings also apply to NCRE. Note that these findings have not been reported in the previous study [19].

### 2.3. Ultrasound Radiofrequency Signal Processing for Quantitative Ultrasound Imaging

This subsection describes the ultrasound backscatter RF signal processing pipelines for ultrasound NWCRE imaging, as well as ultrasound NCRE imaging and B-mode imaging (Figure 1). All the signal processing algorithms were implemented using Matlab (version 2020a, The MathWorks, Natick, MA, USA).

Let **S** denote a matrix of ultrasound backscatter RF signals, $S\in {\mathbb{R}}^{m\times n}$ and S≠0, consisting of *m* scan lines (lateral), with *n* sampling points in each scan line (axial). First, the backscatter envelope signal matrix **E**, $E\in {\mathbb{R}}_{+}^{m\times n}$ and E≠0, was obtained via the Hilbert transform:

(12) $$E=\left|S+jH\right|$$

where **H** is the Hilbert transform of **S**, and j is the imaginary unit. Specifically, the Matlab function Hilbert() was employed for envelope detection (Figure 1). The proposed ultrasound NWCRE imaging method consists of the following steps:

Step 1. A set of blocks (red rectangles in Figure 1), each sized ***w*** scan lines (lateral) × *h* sampling points (axial), corresponding to a physical size of ℒ (lateral, in mm) × ℒ (axial, in mm), were automatically generated on the envelope matrix **E**. The number of blocks was *K* (lateral) × *L* (axial), depending on the block size ℒ and the overlap ratio (OR) between adjacent blocks. A smaller ℒ and a smaller OR yield a larger number of blocks, corresponding to a higher parametric imaging resolution. However, a too small ℒ cannot yield a stable parameter estimation, owing to the number of samples in the block being insufficient. In this study, ℒ was set to 1 time the pulse length of the ultrasound transducer to allow both stable parameter estimation and a high parametric imaging resolution [19]. OR was set at 90% × 90% (lateral × axial) to produce a higher parametric imaging resolution than the conventionally used 50% × 50% (lateral × axial).

Step 2. For each block, the local envelope signal matrix $E_{\mathrm{block}}$, $E_{\mathrm{block}}\in {\mathbb{R}}_{+}^{w\times h}$, was reshaped to a signal vector ${E^{\prime }}_{\mathrm{block}}$, ${E^{\prime }}_{\mathrm{block}}\in {\mathbb{R}}_{+}^{wh\times 1}$. ${E^{\prime }}_{\mathrm{block}}$ was normalized by its RMS using Equation (2) to obtain a normalized local envelope signal vector ${E^{\prime \prime }}_{\mathrm{block}}$. A non-parametric CDF estimation method, i.e., the empirical (Kaplan–Meier) CDF estimator [23], was used for estimating the CDF of ${E^{\prime \prime }}_{\mathrm{block}}$, $\hat{F^{\prime }}(.)$ (Matlab function: ecdf()). With $\hat{F^{\prime }}(.)$, the trapezoidal numerical integration method, was employed to compute the integral in Equation (11) and the integral in Equation (3) (with the upper limit of integration set to $\sqrt{N}$) to estimate the NWCRE and NCRE of $E_{\mathrm{block}}$, respectively. In this study, the parallelized computation method [19] was incorporated for parallelized estimation of NWCRE and NCRE from several blocks at the same time, which has been shown to be faster than the conventional sliding window-based parameter estimation. As a result, an NWCRE map and an NCRE map consisting of NWCRE and NCRE estimates from all the blocks were obtained, with each map sized *K* (lateral) × *L* (axial) (Figure 1). In this study, the Matlab function blockproc() was utilized for automatic block generation and parallelized computation.

Step 3. Because the size of the NWCRE and NCRE maps is smaller than that of **S**, the two maps were resized to *m* × *n* using a bicubic interpolation (Matlab function: imresize()). For a curved-, swept-, or phased-array transducer, digital scan conversion (DSC) was performed on the resized maps to obtain the scan-converted NWCRE and NCRE maps, using an in-house Matlab function. Finally, a color mapping (Matlab function: colormap(jet)) was applied to the scan-converted maps to obtain NWCRE and NCRE parametric images (Figure 1).

In addition to the above-mentioned QUS imaging, ultrasound B-mode imaging was also conducted based on the envelope matrix **E** (Figure 1). Specifically, **E** was normalized by its maximum and the same DSC was performed to the normalized envelope matrix for a curved-, swept-, or phased-array transducer. Logarithmic compression with a 40 dB dynamic range was performed to the scan-converted envelope matrix to construct the B-mode image (Matlab function: db()).

### 2.4. Phantom Simulation Experiments

To validate the capability of the proposed ultrasound NWCRE imaging method in characterizing various scatterer concentrations (number densities), phantom simulation experiments were carried out to generate ultrasound backscatter RF signals, using a system-based simulation method [24]. The speed of sound was set at 1540 m/s and the sampling rate at 50 MHz. A 5 MHz Gaussian pulse with a pulse length of 0.89 mm, a bandwidth of 80%, and a beam width of 1.66 mm was used as the incident ultrasound wave. The area of the ultrasound resolution cell was ~1.48 mm^2^. A homogeneous computer phantom P was a two-dimensional matrix with randomly located delta function δ(.) depicting the spatial arrangements of *M* point scatterers:

(13) $$P(x,y)=\sum_{i=1}^{M}\delta (x-x_{i},y-y_{i})$$

Ultrasound RF signals were obtained by the convolution of the incident ultrasound wave with the computer phantom P. The size of the phantom was set at 30 mm (lateral) × 30 mm (axial). Eight kinds of number densities of scatterers were considered: 2, 4, 6, 8, 10, 12, 14, and 16 scatterers/mm^2^, corresponding to around 2.96, 5.92, 8.88, 11.84, 14.80, 17.76, 20.72, and 23.68 scatterers per resolution cell. For each number density, 50 groups of random phantom simulations were conducted. For each group, a matrix of ultrasound RF signals were collected to construct ultrasound NWCRE, NCRE, and B-mode images, using the algorithms in Section 2.3, and the mean value of a whole QUS parametric image was calculated for statistical analysis. In addition, the dynamic ranges of NWCRE and NCRE were computed by DR_NWCRE_ = NWCRE_max_ − NWCRE_min_ and DR_NCRE_ = NCRE_max_ − NCRE_min_, where the subscripts max and min denote the maximum and minimum QUS parameter values in the whole QUS parametric image. In this study, phantom simulation experiments were performed using Matlab (version 2020a, The MathWorks, Natick, MA, USA).

### 2.5. Clinical Experiments

To validate the feasibility of the proposed ultrasound NWCRE imaging method in characterizing hepatic steatosis, clinical liver ultrasound backscatter RF signals used in our previous work [19] were included. The present analysis was retrospective. The dataset used in this study was the same as the previous study [19]. The RF data collection was approved by the Institutional Review Board of Chang Gung Memorial Hospital in Taiwan, with written informed consent obtained from each subject. The collected liver ultrasound RF signals were divided into two groups, group A and group B. In group A, RF signals were collected from 72 liver donors, with the hepatic fat fraction (HFF, in %) measured by magnetic resonance spectroscopy (MRS) taken as the reference standard. Group B involved 204 patients with chronic hepatitis B, with liver biopsy histopathology as the reference standard. For each patient in group B, a liver specimen was obtained from the right liver lobe with an intercostal approach under the guidance of ultrasound B-mode imaging. All liver specimens were placed in formalin and sent to the Department of Pathology, Chang Gung Memorial Hospital at Linkou, Taiwan, for histological analysis. The liver specimens were fixed in paraffin and stained with hematoxylin–eosin, and then read on-site by expert liver pathologists. Those specimens with a minimum of six portal tracts were suitable for histologic evaluation [25]. According to Brunt et al. [26], hepatic steatosis was graded into G0 (steatosis involving < 5% of hepatocytes); G1 (steatosis involving 5–32.99% of hepatocytes); G2 (steatosis involving 33–66% of hepatocytes); and G3 (steatosis involving > 66% of hepatocytes), corresponding to healthy liver; mild hepatic steatosis; moderate hepatic steatosis; and severe hepatic steatosis.

The clinical liver ultrasound RF signals were acquired using an ultrasound scanner (Model 3000, Terason, Burlington, MA, USA) with a Model 5C2A (Terason) curved-array transducer (central frequency: 3 MHz; pulse length: ~2.3 mm). The sampling rate of the ultrasound RF signals was 12 MHz. A matrix of ultrasound RF signals contained 256 scan lines and each scan line had 1247 sampling points. Each matrix of clinical ultrasound RF signals was used to construct liver ultrasound NWCRE, NCRE, and B-mode images, using the algorithms in Section 2.3. For each case, a liver region of interest (ROI) was manually delineated on the ultrasound B-mode image by an expert radiologist with over 15 years’ experience in liver ultrasound. The mean value of a QUS parametric image within the liver ROI was computed for statistical analysis. In addition, the dynamic ranges of NWCRE and NCRE were computed by DR_NWCRE_ = NWCRE_max_ − NWCRE_min_ and DR_NCRE_ = NCRE_max_ − NCRE_min_, where the subscripts max and min denote the maximum and minimum QUS parameter values in the liver ROI.

### 2.6. Statistical Analysis

In this study, all the statistical analysis was performed using Matlab (version 2020a, The MathWorks, Natick, MA, USA). For phantom simulations, a box plot of the mean QUS parameter (NWCRE and NCRE) values within the whole QUS image for various scatterer number densities were generated. Another box plot of the dynamic ranges of NWCRE and NCRE imaging was also provided.

For group A of subjects, the scatter plot between the mean QUS parameter values within the liver ROI and log_10_(HFF) was generated (Matlab function: scatter()), with the Spearman correlation coefficient *r* (*p*-value) calculated (Matlab function: corr()), where log_10_(HFF) was utilized instead of HFF because previous studies showed that QUS parameters are better correlated with log_10_(HFF) than with HFF [19]. In addition, a box plot of the QUS imaging dynamic ranges was generated.

For group B of subjects, a receiver operating characteristic (ROC) analysis was performed and the area under the ROC curve (AUC) was calculated with the 95% confidence interval (CI), to assess the diagnostic performance of NWCRE and NCRE parametric imaging in discriminating various grades of hepatic steatosis. Three binary classifications were performed: G0 versus G1–G3 (≥G1); G0–G1 versus G2–G3 (≥G2); and G0–G2 versus G3 (≥G3). The ROC analysis was performed based on a Matlab function by Cardillo (http://www.mathworks.com/matlabcentral/fileexchange/12705 (5 September 2026)). The DeLong test was conducted to determine whether the AUCs of the two QUS imaging approaches differ significantly, using an in-house Matlab function. In addition, box plots of the mean QUS parameter values within the liver ROI for various steatosis grades and of the QUS imaging dynamic ranges were generated for group B.

To evaluate whether there was a statistically significant difference between the dynamic ranges of ultrasound NWCRE and NCRE imaging, the *p*-value between the two QUS imaging methods was calculated with respect to phantom simulations, group A of subjects, and group B of subjects, respectively. First, normality of data was assessed using the Shapiro–Wilk test for each dynamic range data using a Matlab function by BenSaïda (https://www.mathworks.com/matlabcentral/fileexchange/13964-shapiro-wilk-and-shapiro-francia-normality-tests; accessed on 5 September 2026). Then, the paired *t*-test was used to calculate the *p*-value if both dynamic range data were approximately normally distributed (Matlab function: ttest()); otherwise, the Wilcoxon signed-rank test was adopted for comparison (Matlab function: signrank()).

Statistical significance was defined as *p* < 0.05. We did not apply formal adjustment for multiple comparisons, and all reported *p*-values are nominal raw *p*-values.

## 3. Results

### 3.1. Phantom Simulations

**Figure 2:**
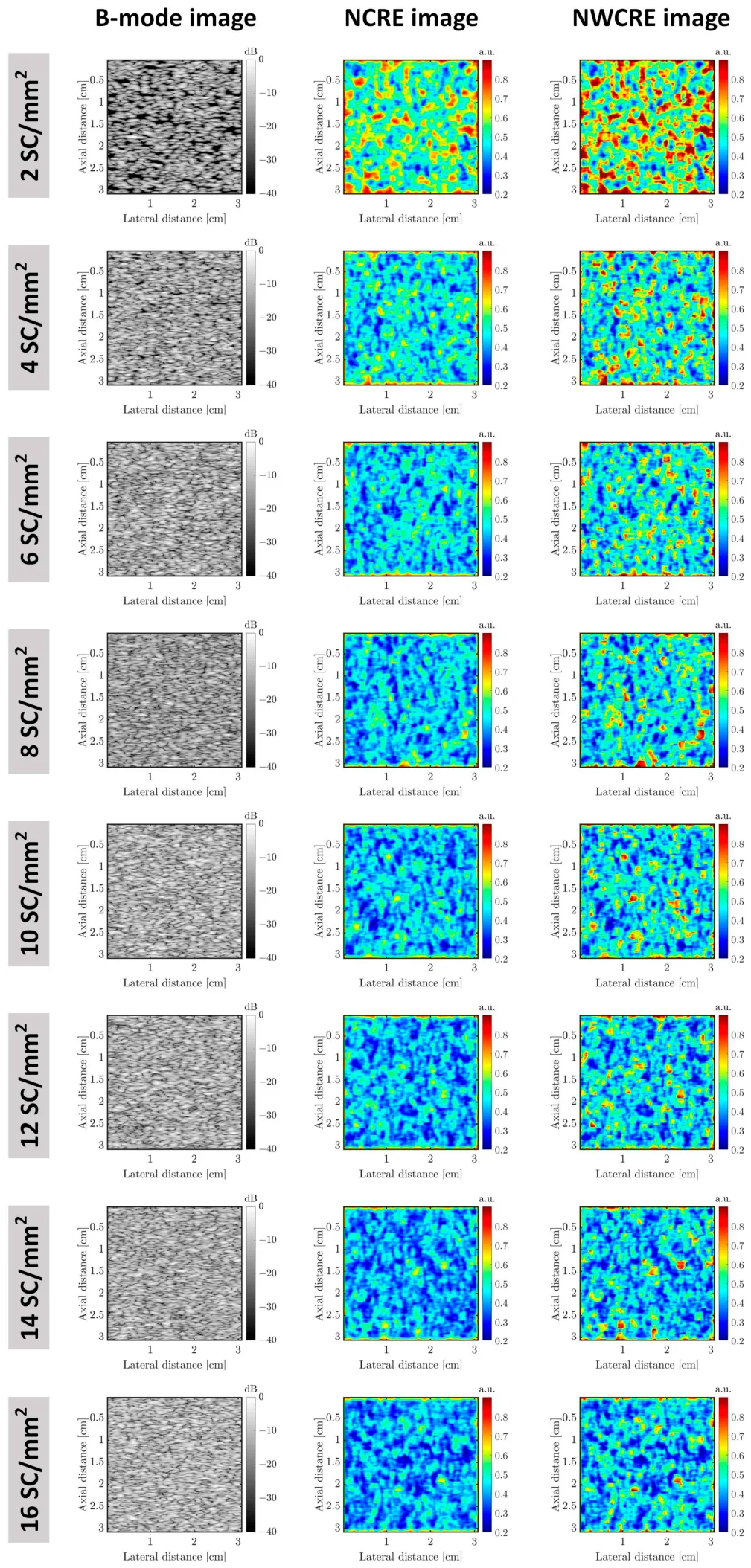
Representative ultrasound B-mode (the first column), NCRE (the second column) and NWCRE (the third column) parametric images corresponding to various scatterer number densities (SC/mm^2^) in phantom simulations. NCRE: normalized cumulative residual entropy; NWCRE: normalized weighted cumulative residual entropy; SC: scatterers.

**Figure 3:**
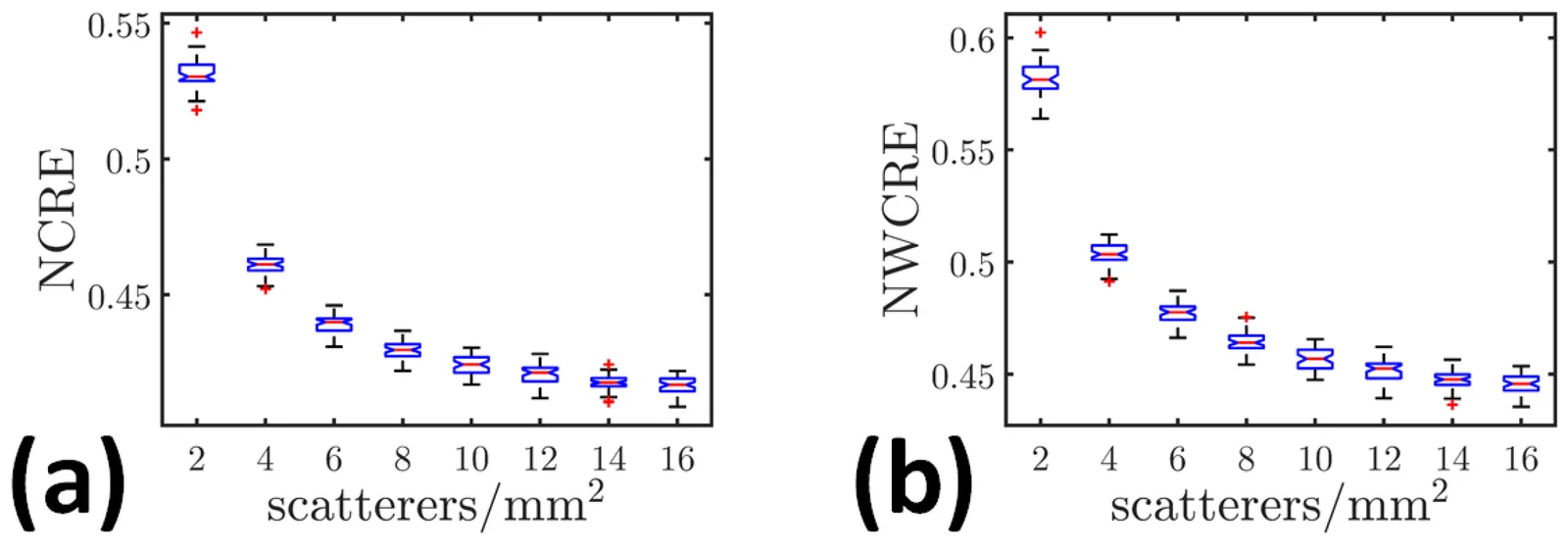
Box plots of the mean NCRE (**a**) and NWCRE (**b**) values corresponding to various scatterer number densities (scatterers/mm^2^) in phantom simulations (*n* = 50 for each number density). For each scatterer number density, 50 two-dimensional computer phantoms were generated, with scatterers randomly distributed, and the ultrasound radiofrequency signals were collected from the phantoms to construct NCRE and NWCRE parametric images. The mean values of NCRE and NWCRE in the whole parametric image were calculated. NCRE: normalized cumulative residual entropy; NWCRE: normalized weighted cumulative residual entropy.

**Figure 4:**
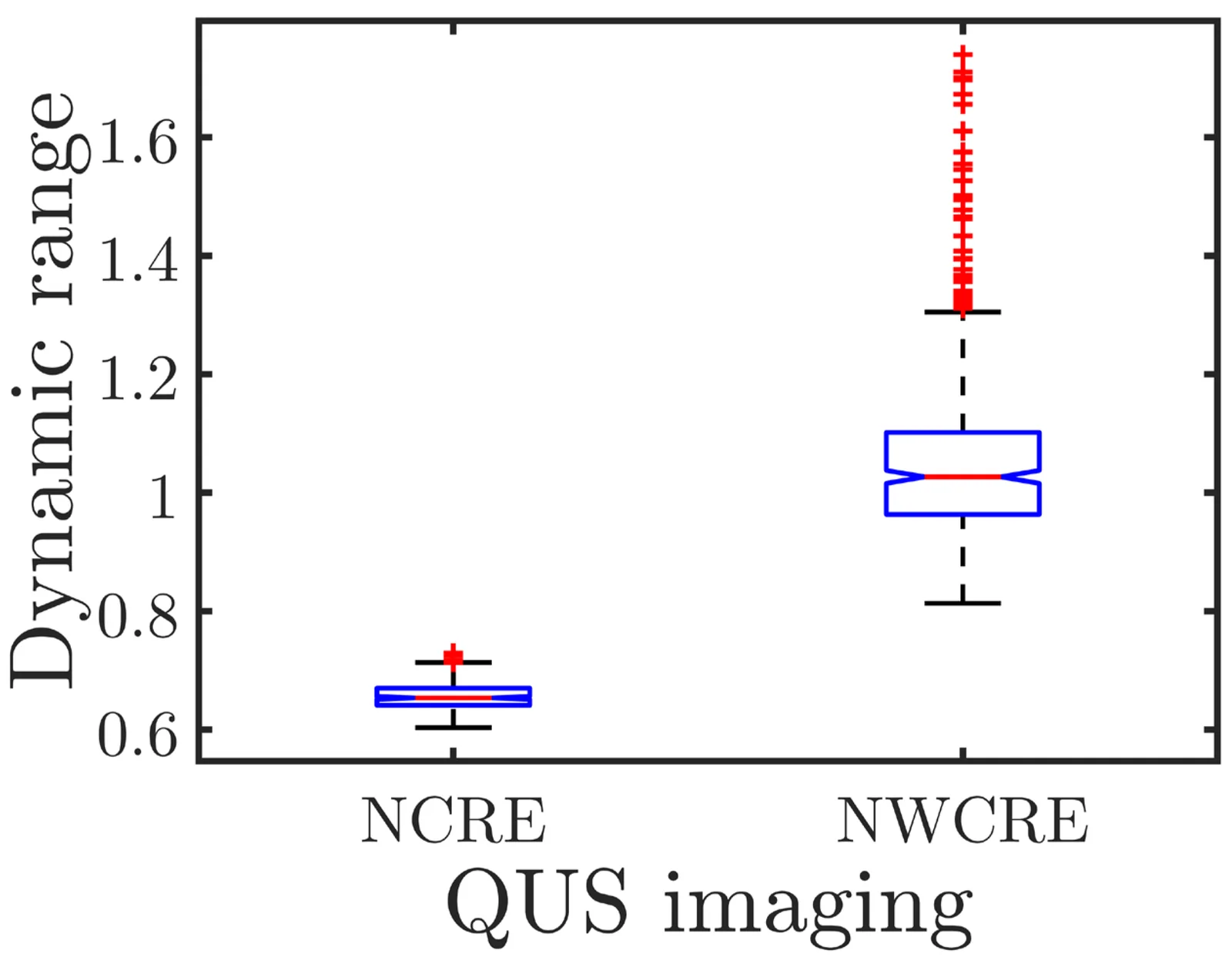
Box plots of the dynamic range of NCRE and NWCRE corresponding to various scatterer number densities (2–16 scatterers/mm^2^) in phantom simulations (*n* = 400). QUS: quantitative ultrasound; NCRE: normalized cumulative residual entropy; NWCRE: normalized weighted cumulative residual entropy.

Figure 2 shows representative ultrasound B-mode as well as NCRE and NWCRE parametric images in phantom simulations, corresponding to various scatterer number densities. With the increase in the scatterer number density, the shadings of NCRE and NWCRE parametric images showed a decreasing trend. Such a trend is quantitatively exhibited in Figure 3. These results demonstrated that the proposed ultrasound NWCRE imaging is capable of characterizing various scatterer number densities. From Figure 2, it was also visually observed that NWCRE images have higher dynamic ranges than NCRE images do for each scatterer number density, which is quantitatively shown in Figure 4. Furthermore, the Wilcoxon signed-rank test shows that the dynamic range of NWCRE imaging is significantly higher than that of NCRE imaging in phantom simulations (*p* < 0.0001).

### 3.2. Clinical Hepatic Steatosis Characterization

**Figure 5:**
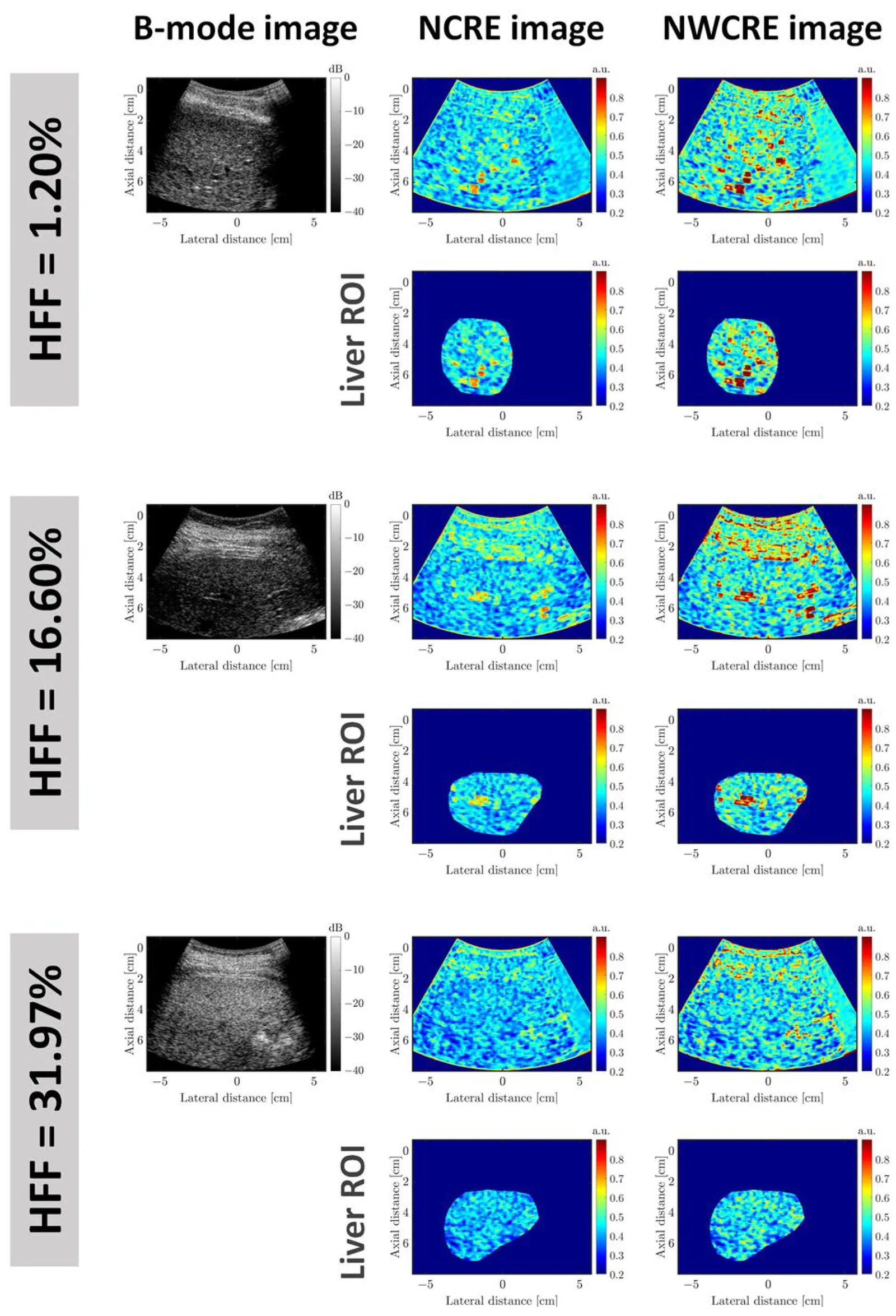
Representative ultrasound B-mode (the first column), NCRE (the second column) and NWCRE (the third column) parametric images and the liver ROI images corresponding to various HFFs in group A. NCRE: normalized cumulative residual entropy; NWCRE: normalized weighted cumulative residual entropy; HFF: hepatic fat fraction; ROI: region of interest; a.u.: arbitrary unit.

**Figure 6:**
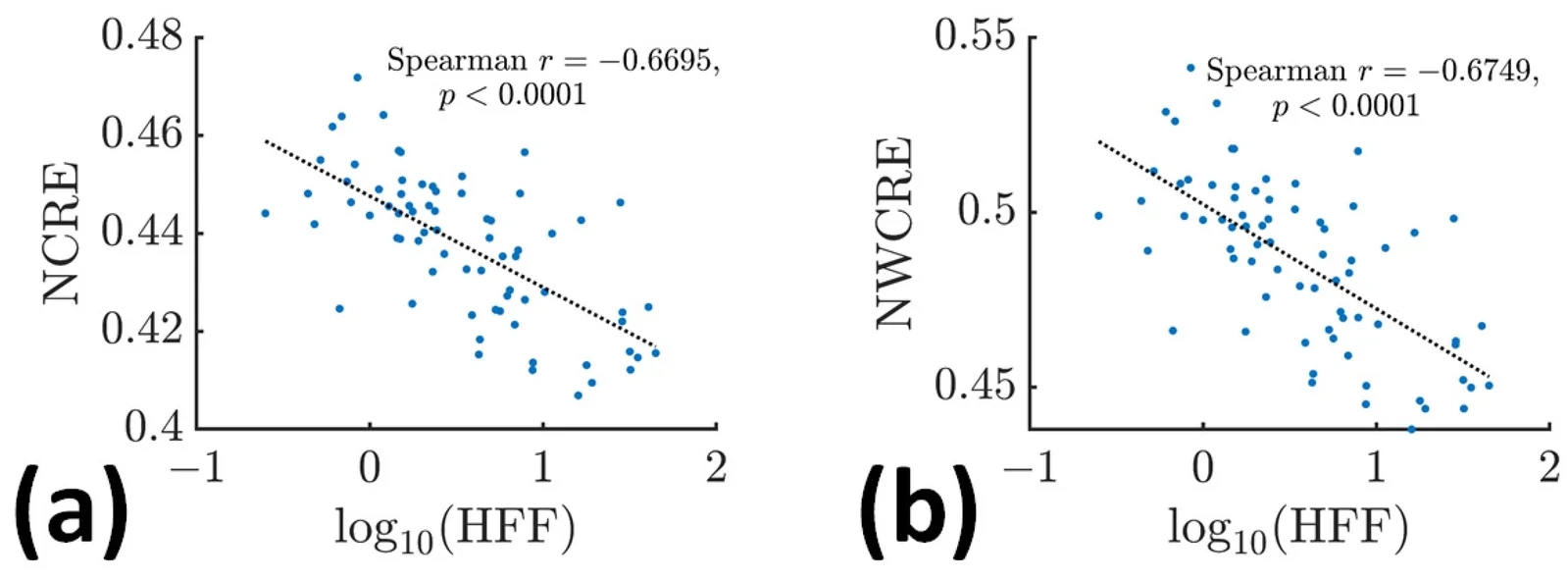
Scatter plots of the mean NCRE (**a**) and NWCRE (**b**) values within the liver regions of interest as a function of log_10_(HFF) in group A (*n* = 72). The dotted lines correspond to fitting lines. HFF: hepatic fat fraction.

**Figure 7:**
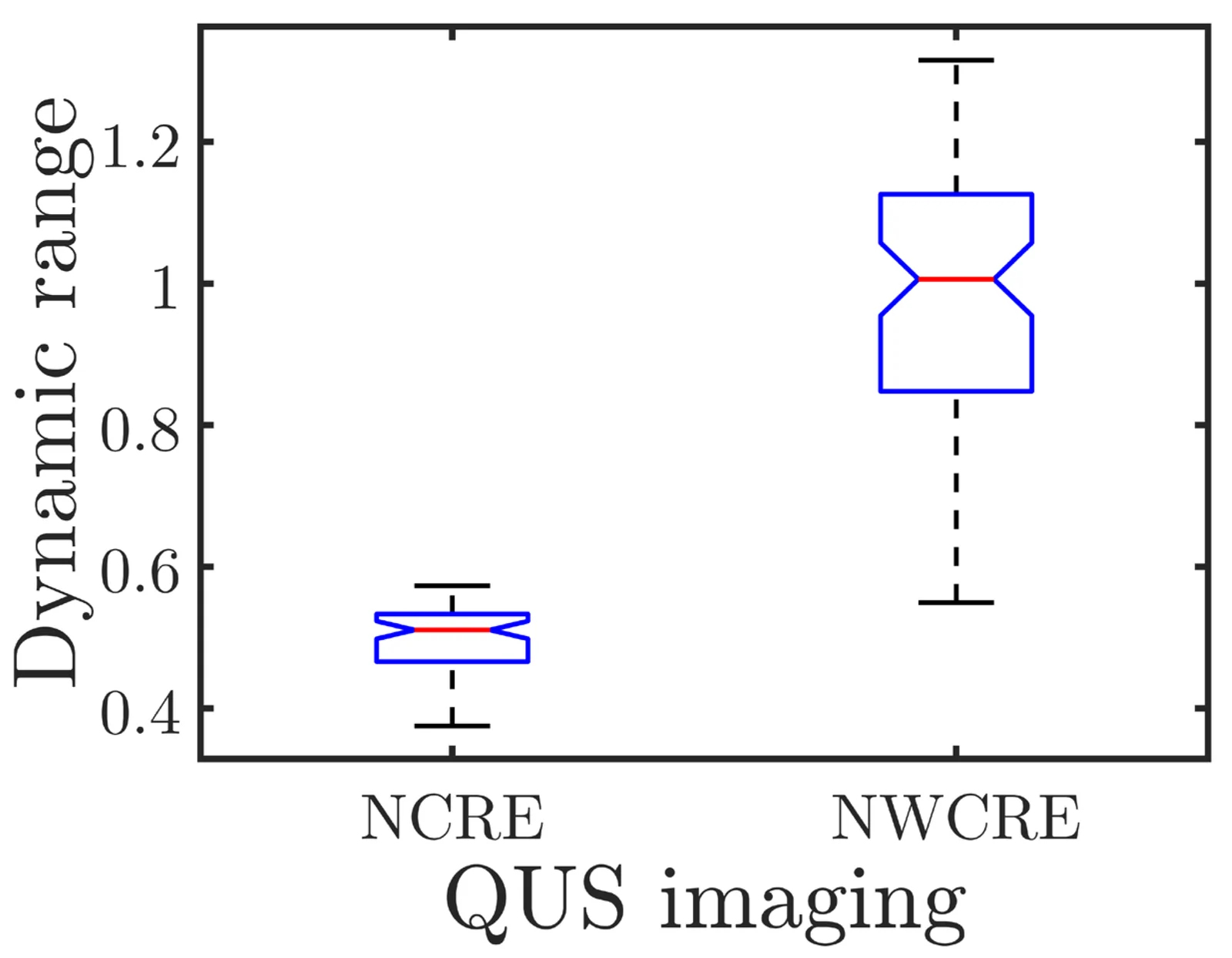
Box plots of the dynamic range of NCRE and NWCRE within the liver regions of interest in group A (*n* = 72). QUS: quantitative ultrasound; NCRE: normalized cumulative residual entropy; NWCRE: normalized weighted cumulative residual entropy.

Figure 5 shows representative ultrasound B-mode as well as NCRE and NWCRE parametric images and the liver ROI images corresponding to various HFFs in group A. As HFF increases, the shadings of NCRE and NWCRE parametric images exhibit a generally declining trend in the liver ROIs, which can be quantitatively observed in Figure 6, wherein scatter plots of the mean NCRE and NWCRE values within the liver ROIs as a function of log_10_(HFF) in group A are shown. Both NCRE and NWCRE are correlated with log_10_(HFF), and NWCRE has a slightly higher absolute correlation coefficient (Spearman *r* = −0.6749, *p* < 0.0001) with log_10_(HFF) than NCRE (Spearman *r* = −0.6695, *p* < 0.0001). In addition, it can be visually observed from Figure 5 and quantitatively observed from Figure 7 that NWCRE imaging had a higher dynamic range than NCRE imaging. Moreover, the Wilcoxon signed-rank test shows that the dynamic range of NWCRE imaging is significantly higher than that of NCRE imaging in group A of subjects (*p* < 0.0001).

**Figure 8:**
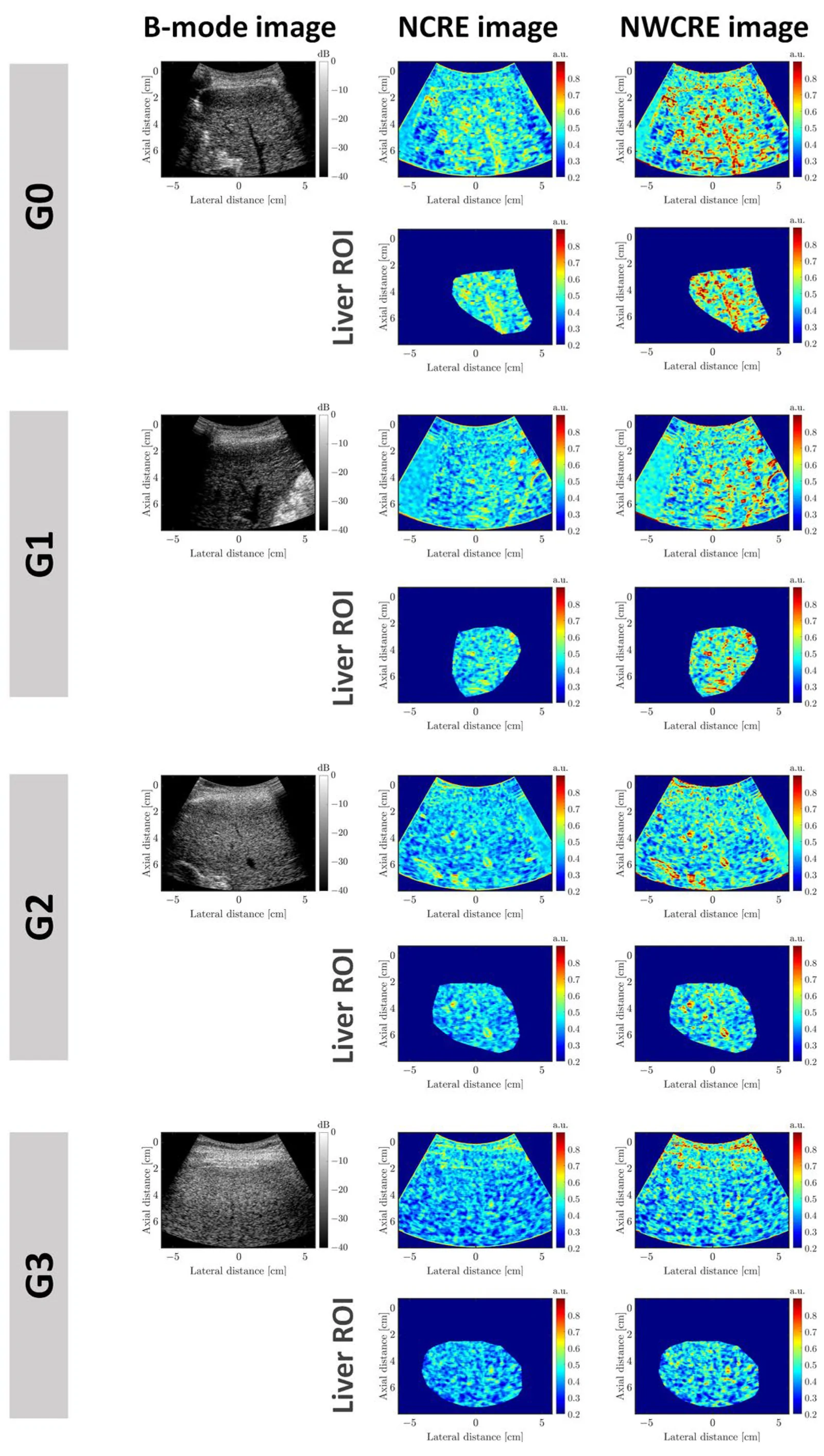
Representative ultrasound B-mode (the first column), NCRE (the second column) and NWCRE (the third column) parametric images and the liver ROI images corresponding to various hepatic steatosis grades in group B. NCRE: normalized cumulative residual entropy; NWCRE: normalized weighted cumulative residual entropy; ROI: region of interest; a.u.: arbitrary unit.

**Figure 9:**
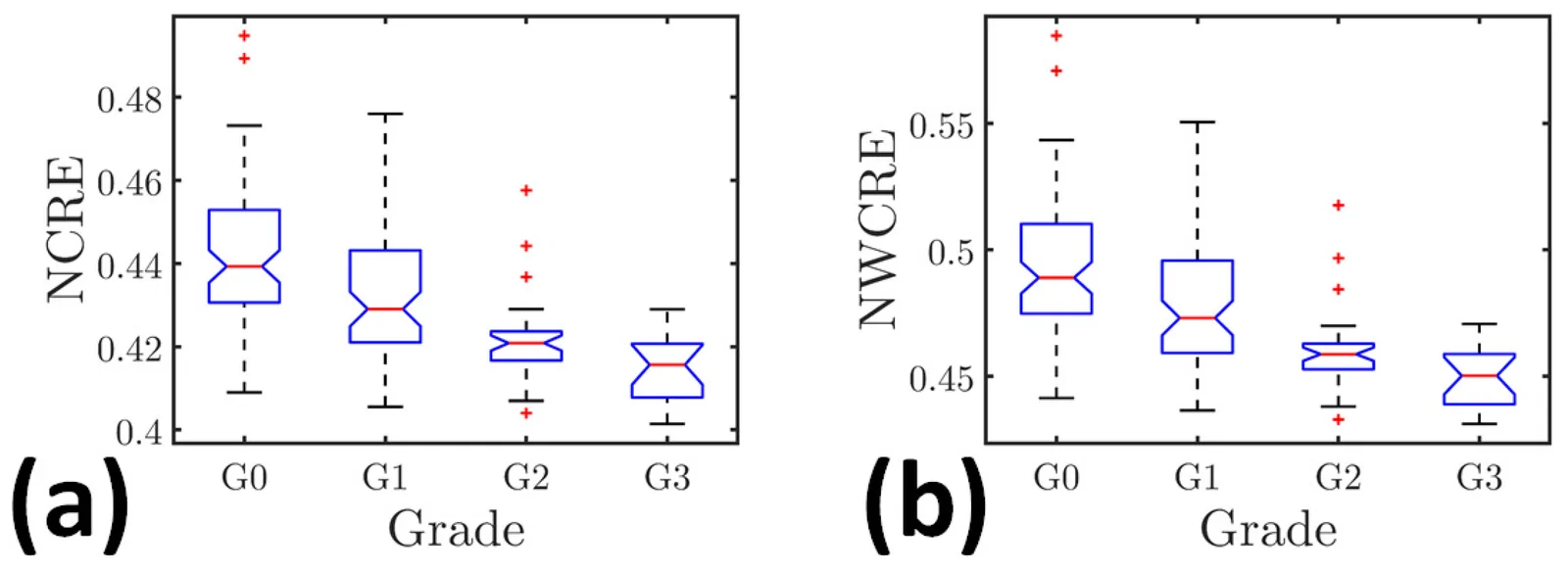
Box plots of the mean NCRE (**a**) and NWCRE (**b**) values within the liver regions of interest corresponding to each steatosis grade in group B (G0, *n* = 80; G1, *n* = 70; G2, *n* = 36; G3, *n* = 18). NCRE: normalized cumulative residual entropy; NWCRE: normalized weighted cumulative residual entropy.

**Figure 10:**
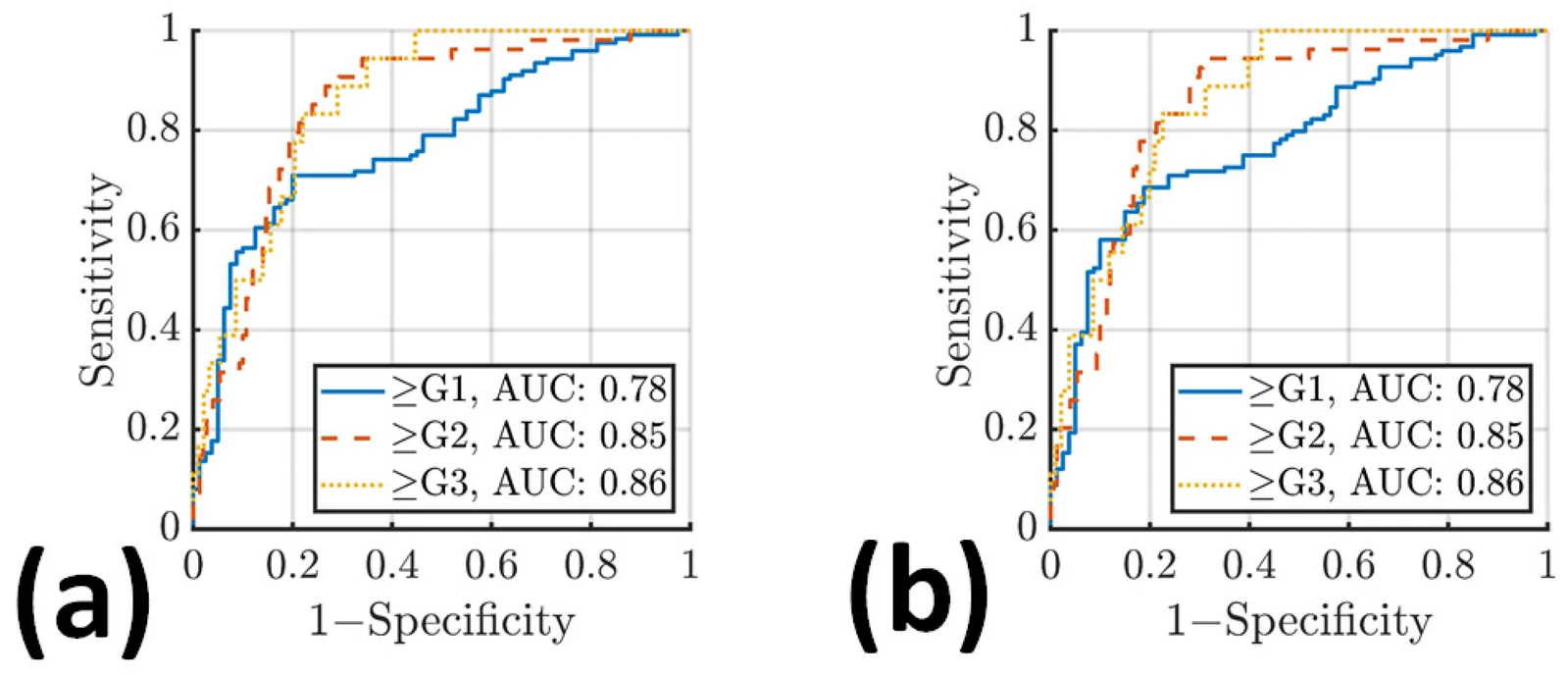
ROC curves for diagnosing various grades of hepatic steatosis using ultrasound NCRE (**a**) and NWCRE (**b**) parametric imaging in group B (G0, *n* = 80; G1, *n* = 70; G2, *n* = 36; G3, *n* = 18). ROC: receiver operating characteristic; AUC: area under the ROC curve.

**Figure 11:**
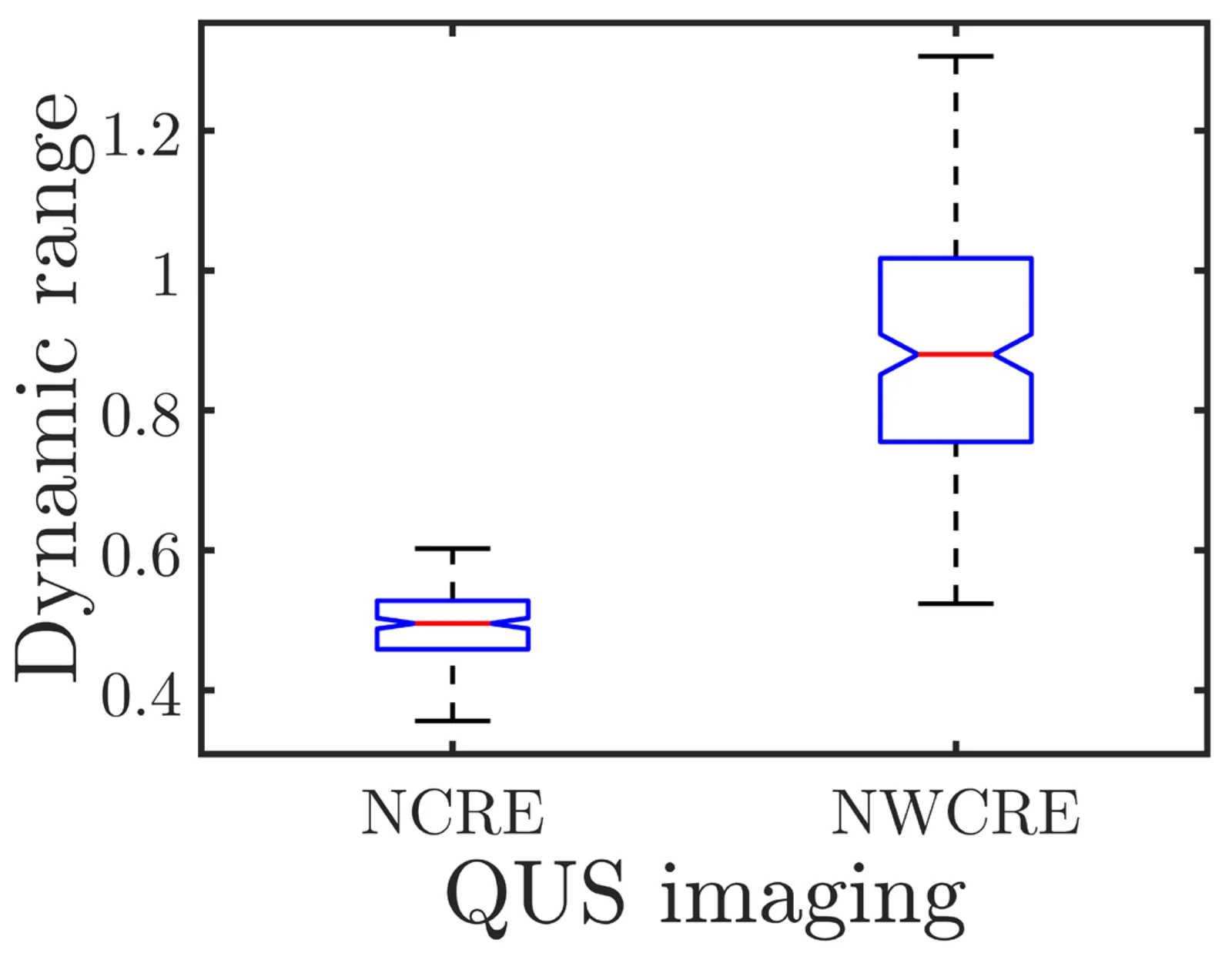
Box plots of the dynamic range of NCRE and NWCRE within the liver regions of interest in group B (*n* = 204). QUS: quantitative ultrasound; ROC: receiver operating characteristic; AUC: area under the ROC curve.

For group B of subjects, liver biopsy histopathology reveals that the number of subjects in G0, G1, G2, and G3 are 80, 70, 36, and 18, respectively. Figure 8 shows representative ultrasound B-mode as well as NCRE and NWCRE parametric images and the liver ROI images corresponding to various hepatic steatosis grades in group B. As the hepatic steatosis grade rose from G0 to G3, the NCRE and NWCRE image shadings generally decreased, similar to those in group A, which can be quantitatively observed in Figure 9. Figure 10 shows ROC curves for diagnosing various grades of hepatic steatosis using NCRE and NWCRE parametric imaging in group B. The AUCs for diagnosing hepatic steatosis grade ≥ G1, ≥G2, and ≥G3 using NCRE and NWCRE imaging were both 0.78, 0.85, and 0.86. The DeLong test revealed no significant AUC differences between NCRE and NWCRE when distinguishing G0 from G1–G3 (*p* = 0.7956), G0–G1 from G2–G3 (*p* = 0.7296), and G0–G2 from G3 (*p* = 0.8330). Both NCRE and NWCRE imaging methods were capable to diagnose various hepatic steatosis grades. Table 1 compares the diagnostic performance between NWCRE and NCRE imaging in grading hepatic steatosis in group B. It can be seen that the diagnostic performance of NWCRE imaging is comparable to that of NCRE imaging. However, it can be visually observed from Figure 8 and quantitatively observed from Figure 11 that NWCRE imaging had a higher dynamic range than NCRE imaging. Moreover, the Wilcoxon signed-rank test shows that the dynamic range of NWCRE imaging is significantly higher than that of NCRE imaging in group B of subjects (*p* < 0.0001).

In the clinical experiments, the Matlab average running time for obtaining an NWCRE map from a frame of uncompressed envelopes was 3.26 ± 0.35 s using the parallelized computation method, compared to 11.06 ± 0.87 s using the conventional for-loop-based computation method, both for five repetitions. The Matlab average running time for obtaining an NCRE map from a frame of uncompressed envelopes was 2.16 ± 0.23 s using the parallelized computation method, compared to 10.95 ± 0.81 s using the conventional for-loop-based computation method, both for five repetitions. The acceleration by the parallelized computation method is approximately 3.4-fold for NWCRE and 5.1-fold for NCRE. The configuration of the personal computer for the above time efficiency analysis was Intel(R) Xeon(R) W-2223 CPU (4-core, 8-thread) @ 3.60 GHz and 32 GB RAM.

## 4. Discussion

In this study, we proposed ultrasound NWCRE imaging for hepatic steatosis characterization and integrated parallelized computation to accelerate parametric map generation. To our knowledge, this is the first study to introduce information-theoretic WCRE to the QUS domain. Moreover, we mathematically deduced the finite upper integration bound for both NWCRE and NCRE, converting original improper integrals into computable proper integrals; this theoretical derivation has not been reported in prior related studies. Phantom simulations and clinical liver ultrasound experiments were performed to demonstrate the proposed ultrasound NWCRE imaging method in characterizing various scatterer number densities and hepatic steatosis grades. The proposed parallelized ultrasound NWCRE imaging yields diagnostic performance comparable to NCRE imaging (all *p* > 0.05 for AUC comparison) and improves the dynamic range over NCRE imaging.

Compared with model-based QUS envelope statistics approaches such as ultrasound Nakagami imaging [6,7,8,9] and homodyned-K imaging [10,11,12,13], the proposed ultrasound NWCRE imaging method does not require the local envelopes to follow specific statistical distributions and allows for a smaller block size for parameter estimation [19] with a higher parametric imaging resolution. Conventional histogram-based ultrasound Shannon entropy imaging estimates the PDF from backscatter RF envelopes [14,15,16,17,18]. Its entropy estimates are highly sensitive to histogram configuration parameters such as bin width and bin count [14,15,16,17,18], which introduces variability in parameter estimation. In contrast, our NWCRE imaging relies on non-parametric empirical CDF estimation rather than PDF histogram fitting, thereby removing the dependence on histogram tuning parameters. Compared with NCRE imaging, NWCRE preserves similar correlation with HFF and equivalent diagnostic performance for hepatic steatosis grading, while delivering significantly higher imaging dynamic range in phantom simulations and clinical data.

Higher dynamic range facilitates better visual contrast within parametric images, which may be beneficial for observing intra-liver heterogeneity of steatosis in clinical practice. Note that in this study and the previous study [19], only the mean value of NWCRE and NCRE parameters were used to analyze the diagnostic performance in grading hepatic steatosis, without considering the intra-liver heterogeneity of these QUS parameter values. When incorporating higher-order statistics of the QUS parameters in future work, with radiomics or deep learning techniques applied to the QUS images, the higher dynamic range of the proposed NWCRE imaging may produce improved diagnostic performance, as it can better reflect intra-liver heterogeneity. However, the incremental clinical value of this higher dynamic range remains to be demonstrated in future studies.

From an acoustic tissue-modeling perspective, normal liver is composed of quasi-regularly distributed acoustic scatterers primarily corresponding to hepatic lobules [6,19]. In hepatic steatosis, abundant large lipid droplets accumulate within hepatocytes, altering the spatial distribution and number densities of effective acoustic scatterers [6,19]. Increased scatterer densities elevate the complexity and uncertainty of backscatter ultrasound RF signals [19]. As scatterer number densities rise, both NWCRE and NCRE values decrease, which is consistent with phantom and clinical observations: higher steatosis severity corresponds to reduced NWCRE and NCRE values. This supports the physical interpretability of CDF-based entropy imaging for characterizing microstructural changes in fatty liver [19].

The key objectives of this study were to introduce information-theoretic WCRE into the QUS field and to investigate the feasibility of the proposed NWCRE imaging method in characterizing tissue microstructures through phantom simulation and clinical hepatic steatosis experiments. However, it should be noted that the proposed NWCRE imaging workflow may be adapted for general ultrasound tissue characterization beyond hepatic steatosis, as it responds to microstructural alterations of scatterers. It is also compatible with existing clinical ultrasound hardware and can serve as an auxiliary quantitative tool complementing conventional B-mode ultrasonography.

This work has several limitations. First, all clinical RF data were acquired using a single ultrasound scanner model. System-related factors including diffraction, transducer response and system gain were not explicitly compensated, so absolute NWCRE values may vary across different ultrasound platforms. In addition, the liver ROIs were delineated manually by only one radiologist, and intra-observer or inter-observer variability (reproducibility) was not evaluated. Second, although NWCRE provides higher dynamic ranges, its overall diagnostic AUC and correlation coefficients with steatosis severity are only comparable relative to NCRE; the gain in diagnostic performance is limited. Third, group B only enrolled patients with chronic hepatitis B, and further validation in general-population MAFLD cohorts is required.

In future studies, the reference phantom method [27] can be applied to mitigate system-dependent biases and enhance cross-system reproducibility. Radiomic features extracted from NWCRE parametric images or deep learning may be combined to further boost diagnostic performance for differentiating steatosis grades. External validation using different ultrasound systems, multiple centers, and broader MAFLD populations is required before clinical generalization.

## 5. Conclusions

In this study, we proposed a novel QUS method termed ultrasound NWCRE imaging for hepatic steatosis assessment. We derived the finite upper limit of integration for NWCRE and NCRE, and incorporated parallelized computation to boost algorithm efficiency. Phantom simulation experiments demonstrated that NWCRE values decrease with increasing scatterer number densities. In clinical validation, NWCRE imaging achieved comparable diagnostic performance for hepatic steatosis grading relative to NCRE imaging (all *p* > 0.05 for AUC comparison), while offering statistically significantly higher imaging dynamic range across phantom and clinical datasets. Nevertheless, the incremental clinical value of this higher dynamic range remains to be demonstrated in future studies. As a CDF-based entropy imaging approach free from histogram-parameter dependency, parallelized NWCRE imaging is a potential QUS technique for characterizing hepatic steatosis.

## Figures and Tables

**Table 1:** Performance of ultrasound NCRE and NWCRE parametric imaging in diagnosing various grades of hepatic steatosis in group B.

Parameter	≥G1		≥G2		≥G3	
	NCRE	NWCRE	NCRE	NWCRE	NCRE	NWCRE
AUC, 95% CI	0.71–0.84	0.71–0.84	0.78–0.92	0.78–0.92	0.75–0.97	0.75–0.97
AUC	0.78	0.78	0.85	0.85	0.86	0.86

Note: NCRE: normalized cumulative residual entropy; NWCRE: normalized weighted cumulative residual entropy; AUC: area under the receiver operating characteristic curve; CI: confidence interval.

## Data Availability

The datasets presented in this article are not readily available due to privacy issues.
